# Identification of differentially accumulated proteins involved in regulating independent and combined osmosis and cadmium stress response in *Brachypodium* seedling roots

**DOI:** 10.1038/s41598-018-25959-8

**Published:** 2018-05-17

**Authors:** Ziyan Chen, Dong Zhu, Jisu Wu, Zhiwei Cheng, Xing Yan, Xiong Deng, Yueming Yan

**Affiliations:** 1 College of Life Science, Capital Normal University, 100048 Beijing, China; 2 Joint Center for Global Change Studies (JCGCS), 100875 Beijing, China

## Abstract

In this study, we aimed to identify differentially accumulated proteins (DAPs) involved in PEG mock osmotic stress, cadmium (Cd^2+^) stress, and their combined stress responses in *Brachypodium distachyon* seedling roots. The results showed that combined PEG and Cd^2+^ stresses had more significant effects on *Brachypodium* seedling root growth, physiological traits, and ultrastructures when compared with each individual stress. Totally, 106 DAPs were identified that are responsive to individual and combined stresses in roots. These DAPs were mainly involved in energy metabolism, detoxification and stress defense and protein metabolism. Principal component analysis revealed that DAPs from Cd^2+^ and combined stress treatments were grouped closer than those from osmotic stress treatment, indicating that Cd^2+^ and combined stresses had more severe influences on the root proteome than osmotic stress alone. Protein–protein interaction analyses highlighted a 14-3-3 centered sub-network that synergistically responded to osmotic and Cd^2+^ stresses and their combined stresses. Quantitative real-time polymerase chain reaction (qRT-PCR) analysis of 14 key DAP genes revealed that most genes showed consistency between transcriptional and translational expression patterns. A putative pathway of proteome metabolic changes in *Brachypodium* seedling roots under different stresses was proposed, which revealed a complicated synergetic responsive network of plant roots to adverse environments.

## Introduction

Cadmium (Cd) and drought are two common abiotic stresses, particularly in places contaminated with heavy metals. The simultaneous occurrence of Cd and drought seriously affects crop growth and yield formation^1,2^. Cd is a highly toxic heavy metal and a widespread environmental pollutant. It can be released into aquatic systems through various industrial and anthropogenic activities as well as geochemical weathering of rocks and other environmental problems like volcanic eruptions, acid rain, and continental dusts, which can be absorbed and accumulated by crops. A large amount of Cd^2+^ not only constrains yields but also eventually results in human health problems through Cd^2+^ accumulation from the food chain^3^. Humans are more affected by Cd^2+^ toxicity than animals due to their longevity and the 15–20-year half-life of Cd^2+^ in humans^4^. For instance, in the 1950s and 1960s in Japan, Cd-contaminated rice led to renal impairment and bone disease in exposed populations^5^. In recent years, rice has been increasingly exposed to heavy metal pollution in the environment and the contamination of Cd^2+^ in rice is serious, particularly in China^4,6,7^.

Roots are important organs in plants that uptake water and nutrients from soil, and perceive and transduce various soil signals to shoots, which induce a suite of physiological, morphological, and molecular responses in the whole plant^8^. When subjected to various abiotic stresses from soil, such as drought and heavy metals, roots are the first site for sensing stress signals and initiating signaling cascades at the molecular level in response to adverse environments. Through these responses, plants can regulate gene expression of regulatory and functional proteins to enhance stress tolerance^9^. Plant response to drought stress has become highly important in current plant biology research because drought stress can lead to a complex process that involves morphological, physiological, and biochemical changes^10–13^. When plants suffer from osmotic stress, mitochondrial respiration is reduced by injuring metabolism and altering electron transport, which generate reactive oxygen species (ROS), such as superoxide and peroxides, which have severe toxic effects on plants. ROS can influence the cellular metabolism and cause chemical damage to DNA and proteins^14,15^. Plants have developed a sophisticated and elaborate system for scavenging high levels of ROS using antioxidant enzymes such as superoxide dismutase (SOD), ascorbate peroxidase (APX), peroxidase, and catalase and using antioxidant compounds, including ascorbate and glutathione^16–18^. Moreover, osmotic stress leads to changes in concentrations of plant calcium (Ca^2+^), which can trigger signaling cascades^19^.

Among all heavy metals, Cd is of particular concern due to its severe phytotoxic effects on plants, as they can take up Cd^2+^ easily, contributing to bio-concentration^11,20^. Cd^2+^ can arrest biomass accumulation, influence chloroplasts, photosynthesis, water relations, respiration, and the uptake and translocation of mineral nutrients in plants, thus giving rise to severe growth alterations^21^. Exposure of plants to Cd^2+^ results in oxidative stress at the cellular level, as revealed by accumulation of hydrogen peroxide (H_2_O_2_), lipid peroxidation, and oxidative burst. In plants, the effective functioning of a complex defense system, including metal-binding compounds, low molecular antioxidants, antioxidative enzymes, and those participating in detoxification processes will enhance the tolerance of heavy metals^22^.

To date, numerous proteomic studies of osmotic stress and heavy metal stress in different plant species have been performed due to the rapid advances in genomics, including *Brassica juncea*^23^, rice^2,24^, poplar^25^, Arabidopsis^26,27^, wheat^28–30^, and soybean^31,32^. However, most of these studies only involved a single stress treatment, and few are concerned with the underlying molecular mechanisms of plant responses to a combination of different abiotic stresses. Thus, it is crucial to realize the synergistic response mechanisms of plants suffering from multiple abiotic stresses, particularly under combined osmotic and heavy metal stresses.

*Brachypodium distachyon* L. is a model plant for economically important crops such as wheat, barley, and several other plants. The genome sequencing and annotation of *B*. *distachyon* accession 21 (Bd21) was completed in 2010^33^, which revealed that *Brachypodium* was much more closely related to wheat and barley than rice, sorghum, or maize^33,34^. In this study, we performed the first comparative proteomic analysis of Bd21 seedling roots under polyethylene glycol (PEG), Cd^2+^, and their combined stresses. Our results from the proteome level provide new evidence for further understanding the molecular mechanisms of the plant abiotic stress response.

## Results

### Physiological and ultrastructural changes induced by three different stress treatments in Bd21 seedling roots

**Figure 1:**
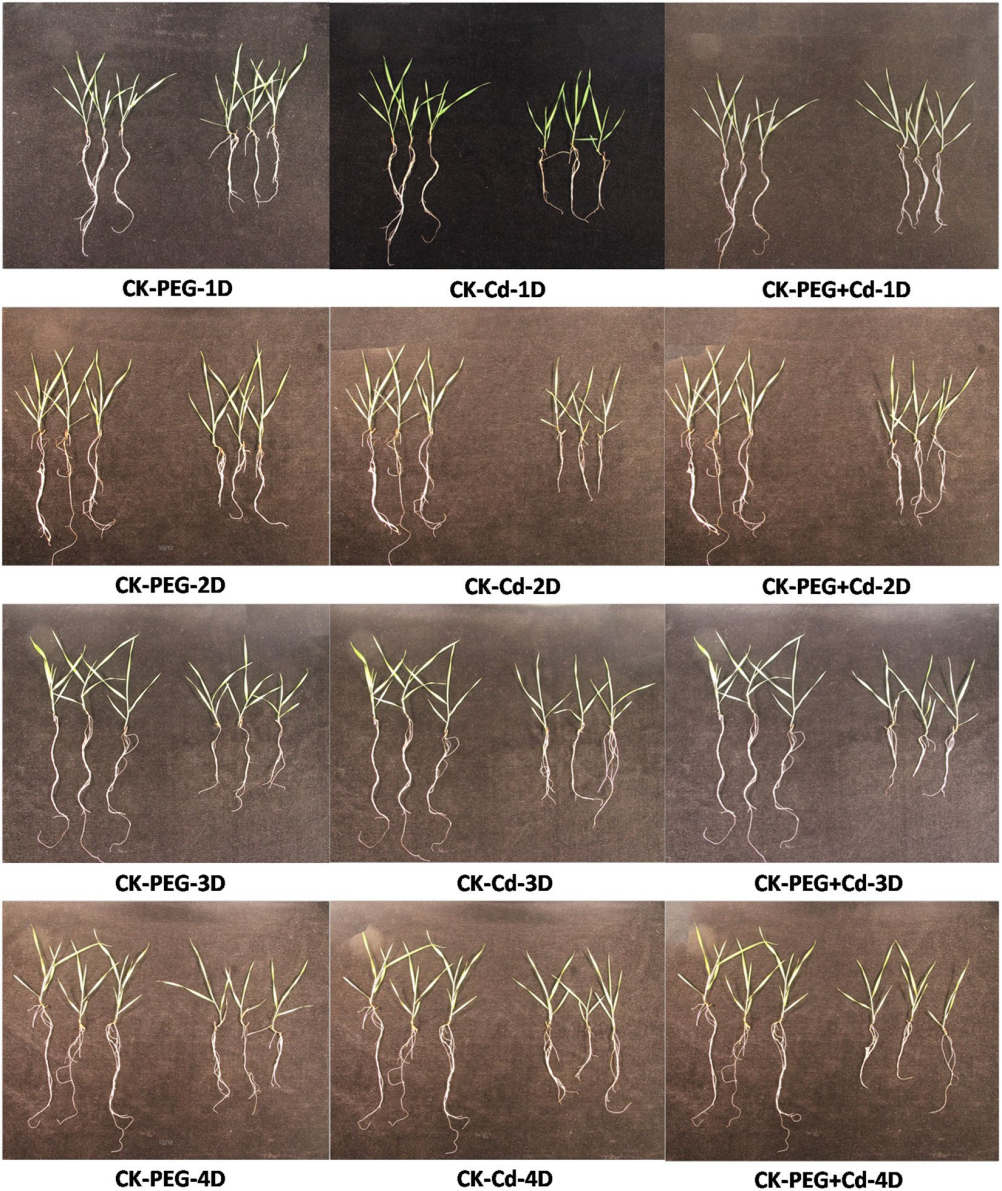
Phenotypic changes of Bd21 seedlings under different stresses.

Observations of seedling phenotypes showed that PEG, Cd^2+^, and their combined stresses significantly affected the growth and development of plants. As shown in Fig. 1, Bd21 seedlings became smaller, their leaves began to curl, and their root tips gradually became darker in color compared with the control over four time periods under the three stress treatments, and the effect was especially significant at 4 days. Moreover, combined stresses had more severe effects than single stress.

**Figure 2:**
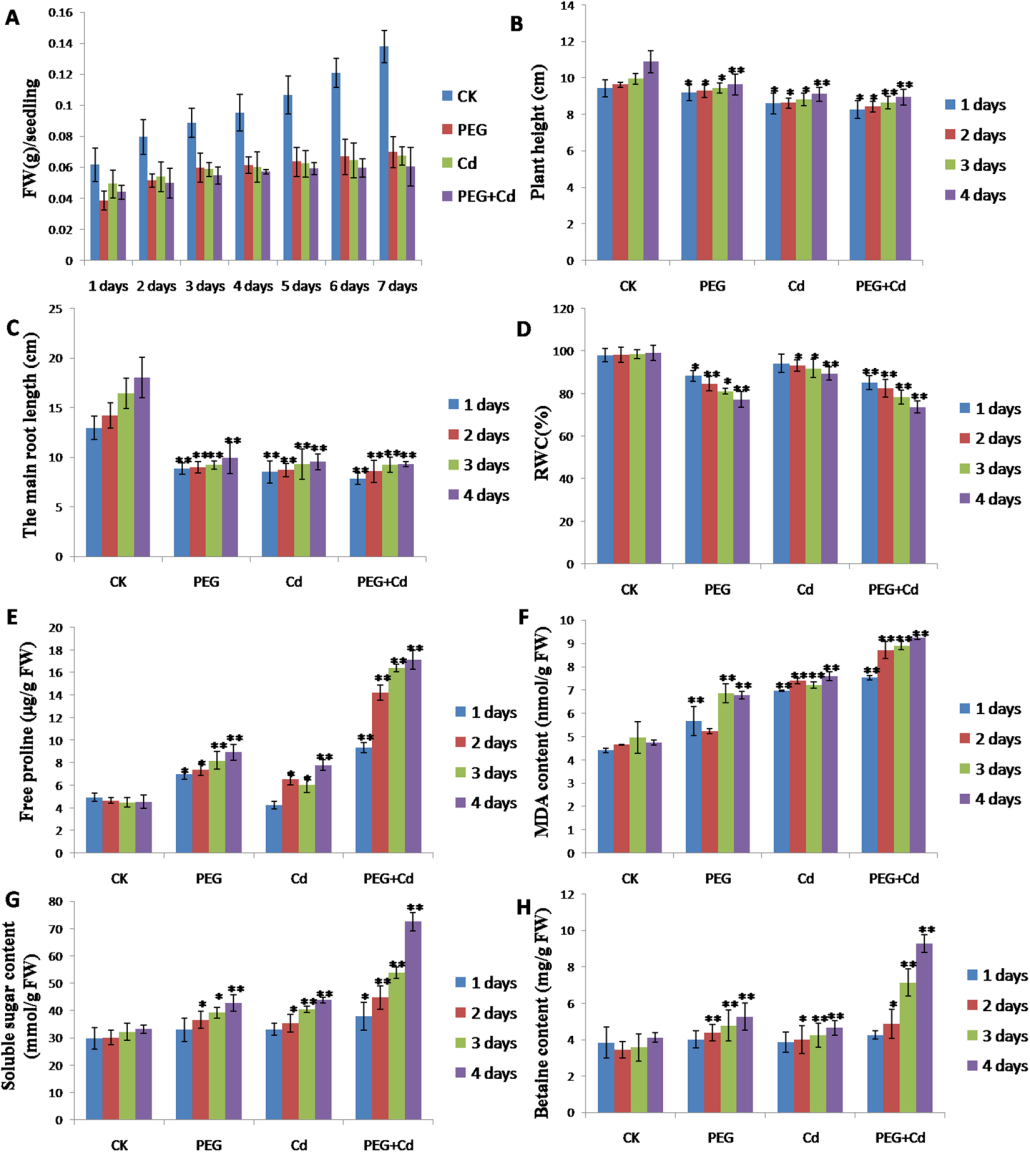
Physiological changes of Bd21 seedling leaves under PEG osmotic stress, Cd^2+^ stress and their combined stress treatments. (**A**) Fresh weight (FW); (**B**) Plant height; (**C**) Main root length; (**D**) Relative water content; (**E**) Proline; (**F**) Malondialdehyde (MDA); (**G**) Soluble sugar; (**H**) Betaine. Error bars indicate standard errors (SE) of three biological replicates. Statistically significant differences between treatments and control were calculated using independent Student’s t-tests: *p < 0.05; **p < 0.01; ***p < 0.001.

Some physiological parameters also showed significant changes under each stress (Fig. 2). Fresh weight (FW) was significantly decreased from 1 to 7 days under the three stress treatments, but after 4 days, FW showed a more significant reduction (Fig. 2A). This suggests that the 4-day treatment was an important node and after that, severe physiological damages to seedlings may occur. Thus, we selected 1–4 day treatments for subsequent physiological parameters and proteome analysis. Seven main physiological indices of seedling roots from four time points (1, 2, 3, and 4 days) were measured to estimate the effects of each treatment on seedling growth (Fig. 2B–H). The results showed that plant height, relative water content, and main root length were significantly reduced under the three stresses, particularly under the combined stresses, whereas proline, malondialdehyde (MDA), soluble sugar, and betaine contents of Bd21 seedling roots were significantly increased, particularly after 4 days of the combined stress treatment.

**Figure 3:**
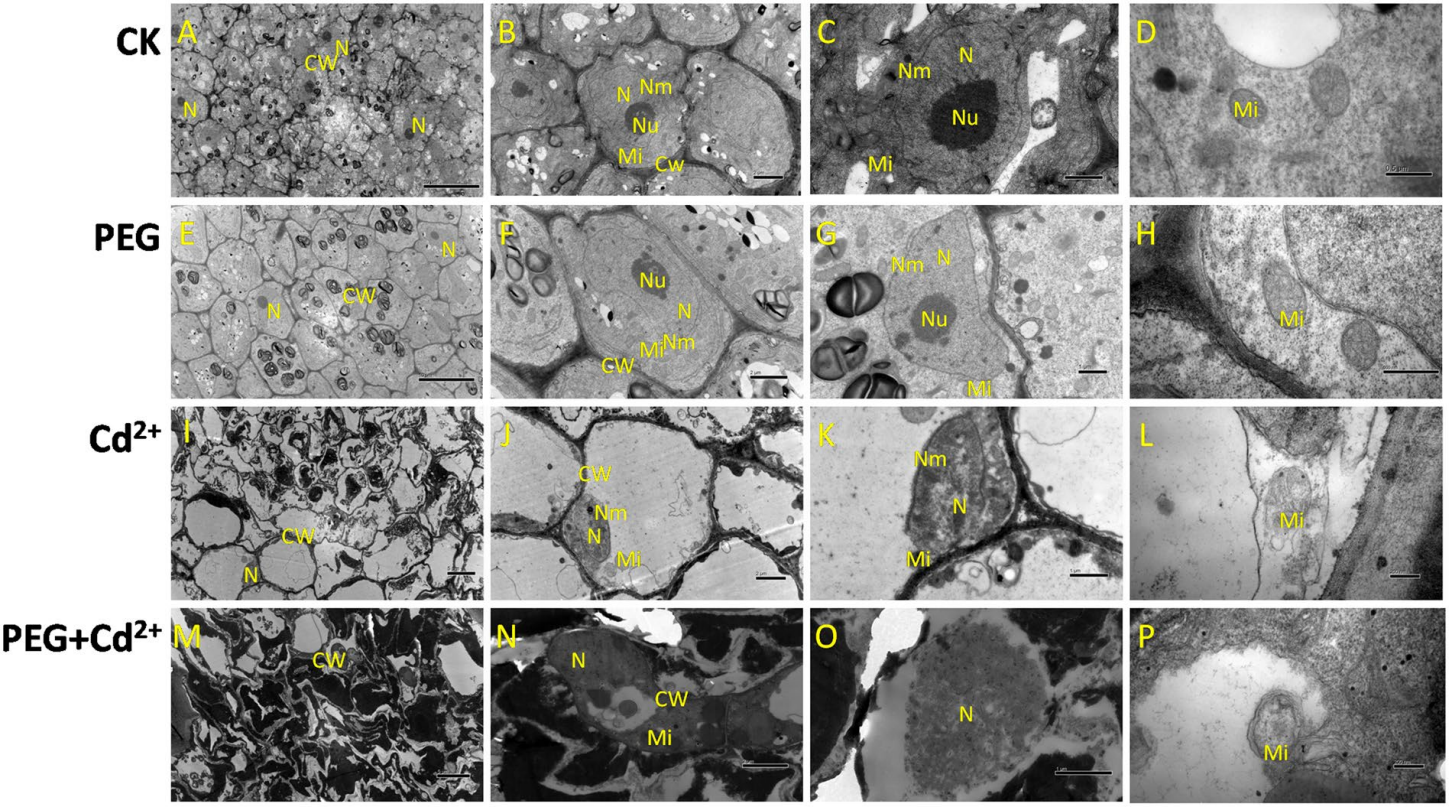
Transmission electron microscope (TEM) images of root ultrastructures under control and stress treatments (2 days). (**A**–**D**): Control; (**E**–**H**): osmotic stress; (**I**–**L**): Cd^2+^ stress; (**M**–**P**): PEG + Cd^2+^ stresses. CW: Cell wall; Mi: Mitochondrion; N: Nucleus; Nm: Nuclear membrane; V: Vacuole.

Further transmission electron microscopy observations indicated that significant cell ultrastructural changes also occurred in seedling roots under the three stress treatments (Fig. 3). The roots of the control had intact structures of root tip cells and normal cell proliferation and division (Fig. 3A,B). Meanwhile, the nucleolus, nuclear membrane, and mitochondria were clearly visible (Fig. 3C,D). Under osmotic stress treatment, the cell membranes were broken and obscured while the cell wall became irregular and broken (Fig. 3E,F). Furthermore, the nucleolus was deformed and spread, and the nuclear membrane was damaged (Fig. 3G). Moreover, the mitochondria were damaged and began to disintegrate and shed the matrix (Fig. 3H). Under Cd^2+^ and combined stress treatments, these phenomena also appeared and cell damage that was more serious than with the single osmotic stress could be observed (Fig. 3I–P).

### Proteome profiles of Bd21 seedling roots in response to different stress treatments

**Figure 4:**
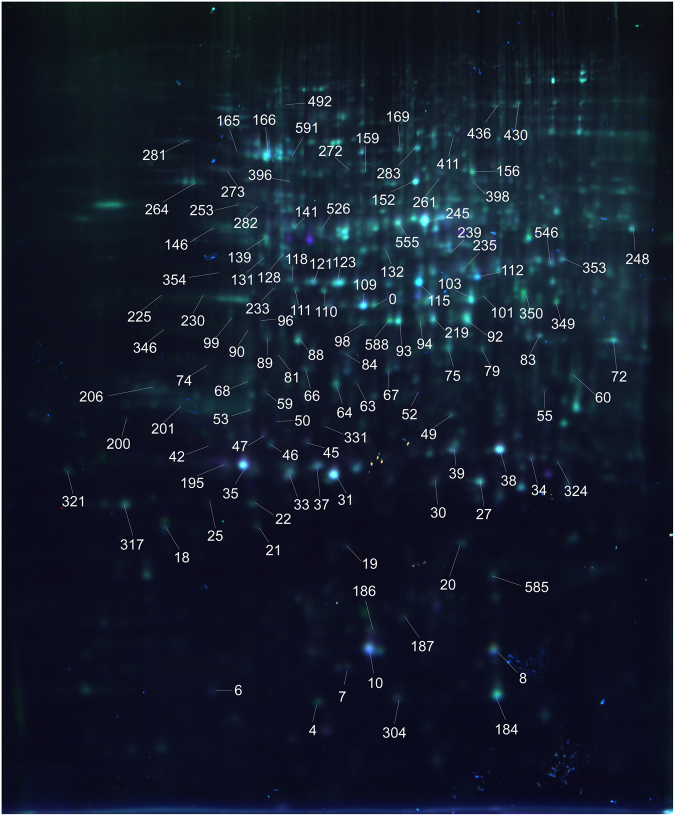
Images from 2D-DIGE analyses of whole proteins from Bd21 seedling roots under different stress treatments. DAP spots on the gel image are numbered.

**Figure 5:**
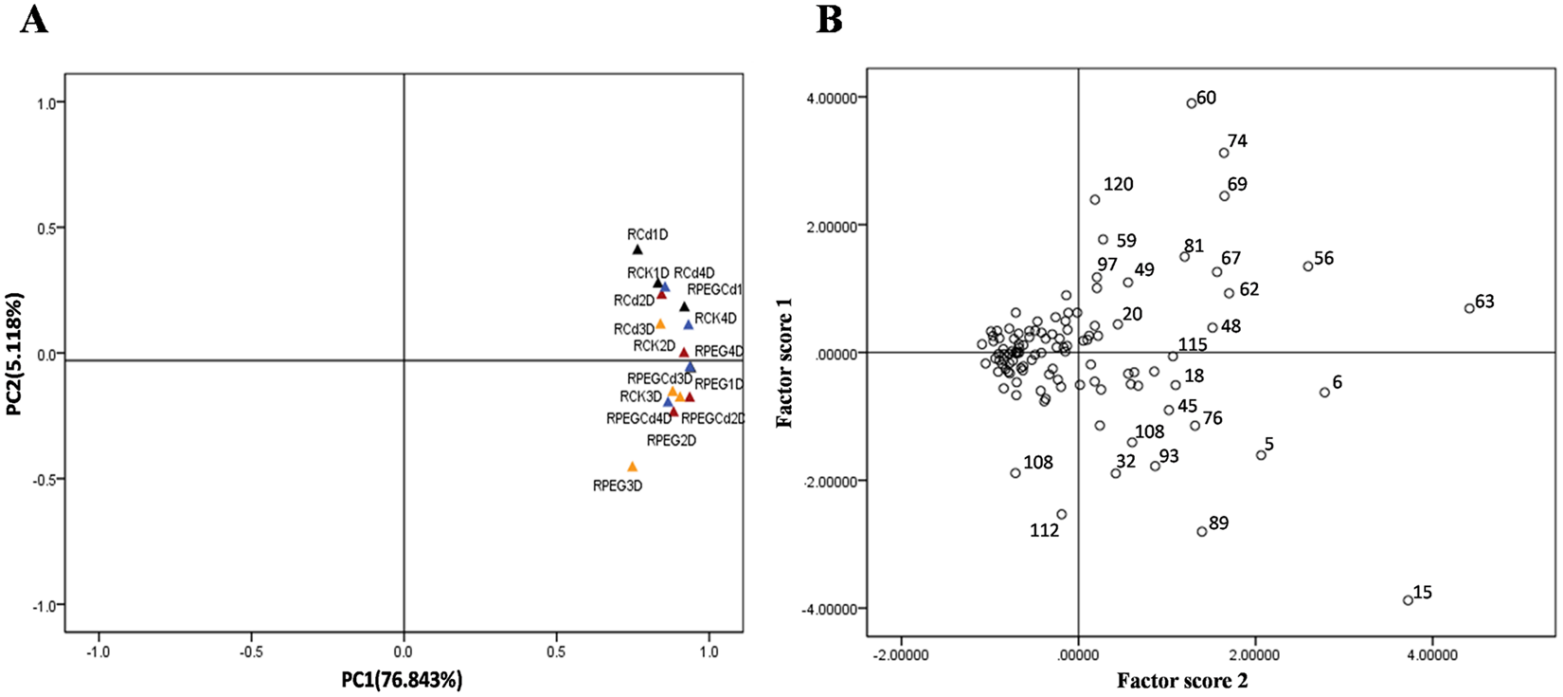
Principal component analysis (PCA) of 172 DAP spots from Bd21 seedling roots under different stress treatments. (**A**) PCA of individual experimental samples. (**B**) Scatter diagram of 172 DAP spots based on PCA.

The differentially accumulated protein (DAP) spots of Bd21 seedling roots under the three treatments after 2 days were identified by two-dimensional difference gel electrophoresis (2D-DIGE) (Fig. 4). Then, two-dimensional electrophoresis (2-DE) was used to separate these DAPs for tandem mass spectrometry and dynamic accumulation profiling analyses at four different treatment times. In total, about 600 protein spots from the roots were reproducibly detected and matched on 2-DE gels. Among them, 135 DAP spots that had at least a twofold difference in abundance were determined and used for principal component analysis (PCA) to identify potential outliers and clusters^12^. PCA can single out the protein species spots that were affected and revealed major differences in protein abundance patterns between control and stress treatments (Fig. 5). It is clear that the samples from the control (CK) (RCK-1d, RCK-2d, RCK-3d, and RCK-4d), osmotic stress treatment (RPEG-1d, RPEG-2d, RPEG-3d, and RPEG-4d), Cd^2+^ stress treatment (RCd-1d, RCd-2d, RCd-3d, and RCd-4d), and combined stress treatment (RPEG-Cd-1d, RPEG-Cd-2d, RPEG-Cd-3d, and RPEG-Cd-4d) were grouped in different PCA plots (Fig. 5A). This indicated an obvious distinction between the control and stress treatments at the proteome level. Particularly, the samples from the Cd^2+^ and combined stresses were much closer to each other when compared with osmotic stress treatment, indicating that the proteome of roots was dramatically altered under Cd^2+^ and combined stresses than PEG stress alone. Furthermore, the spots in roots which show a higher loading with PC2 were proteins related to the type of treatments, PC2 was named as treatment type (Fig. 5A). In addition, some protein species spots were separated from others, which were considered potential outliers and given priority for matrix-assisted laser desorption/ionization time-of-flight/time-of-flight mass spectrometry (MALDI-TOF/TOF-MS) analysis (Fig. 5B). Ultimately, 119 DAP spots were successfully identified by tandem mass spectrometry with a high degree of confidence, which represented 106 unique proteins (Fig. 5 and Tables 1 and S1,2).

**Table 1:** Some key DAP spots in Bd21 seedling roots with significant accumulation changes under three stress treatments.

Spot no.^a^	Protein name	Accession no.^b^	Protein Score^c^	Protein Score C. I.%^d^	Predicted subcellular localisation^e^	Average %vol ratio^f^					
						PEG		Cd		PEG + Cd	
						1 day 3 day	2 day 4 day	1 day 3 day	2 day 4 day	1 day 3 day	2 day 4 day
**Signal transduction:**											
60	Guanine nucleotide-binding protein subunit beta-like protein A (receptor for activated C kinase 1A, RACK1A)	XP_003567944.1	640	100	Cyto	0.9	1.6	0.6	1.3	0.8	1.4
						1.8	0.7	0.7	0.7	1.3	0.4
74	14–3–3-like protein GF14-D	XP_003577480.1	127	100	Cyto	0.6	0.6	0.4	0.7	0.5	0.5
						0.6	0.4	0.7	0.8	0.4	0.5
184	Nucleoside diphosphate kinase II NDPK2	AGK91194.1	545	100	Cyto	0.5	0.6	0.4	0.4	0.7	0.8
						0.7	0.6	0.6	0.5	0.6	0.7
201	14-3-3-like protein A	XP_003560774.1	229	100	Plastid	0.7	0.7	0.8	1.1	1.0	0.7
						0.6	1.3	0.4	0.5	1.1	0.6
206	14–3–3-like protein GF14-B	XP_003579911.1	487	100	Cyto	1.1	1.1	0.8	1.7	0.4	1.6
						0.9	1.2	1.3	1.9	0.8	0.0
**Energy metabolism Energy metabolism**											
31	Triosephosphate isomerase	XP_003568647.1	665	100	Cyto	1.6	1.4	0.6	1.3	1.2	1.9
						1.2	1.4	1.2	0.9	1.4	1.6
33	Triosephosphate isomerase	XP_003568647.1	522	100	Cyto	1.5	1.1	0.8	1.1	1.3	1.3
						0.7	1.2	0.8	1.3	1.3	1.5
45	Probable ATP synthase 24 kDa subunit	XP_003574053.1	199	100	Mito	1.2	1.6	1.6	1.4	0.9	1.6
						1.3	1.3	2.9	1.6	1.5	2.5
72	Glyceraldehyde-3-phosphate dehydrogenase, GAPDH	XP_003573324.1	429	100	Cyto	1.4	0.9	0.7	1.9	2.3	2.5
						1.0	1.3	2.4	1.3	1.7	0.3
92	Malate dehydrogenase, MDH	XP_003574062.1	901	100	Cyto	0.8	0.7	0.5	0.6	0.9	0.7
						0.9	1.1	0.8	1.0	0.8	1.2
152	2,3-bisphosphoglycerate-independent phosphoglyceratemutase-like PGM	XP_003564482.1	681	100	Cyto	1.6	1.8	1.0	1.1	1.3	1.7
						0.8	0.7	1.0	1.1	1.0	0.7
331	Probable ATP synthase 24 kDa subunit	XP_003574053.1	316	100	Mito	2.6	0.9	0	1.2	0.7	0
						3.6	0.7	0	0	1.8	0
526	ATP synthase subunit beta	XP_003569580.1	578	100	Mito	1.2	0.3	0.6	0.8	1.5	0.8
						1.4	1.5	2.3	2.1	0.9	1.2
555	Enolase	XP_003573806.1	418	100	Cyto	3.4	2.9	3.2	5.4	3.8	3.2
						1.2	1.6	1.6	2.8	2.8	5.9
**Protein metabolism**											
18	40 S ribosomal protein S7	XP_003558127.1	151	100	Cyto	1.2	1.4	1.6	1.3	1.1	1.4
						1.0	0.8	1.0	0.9	1.7	2.9
159	Heat shock 70 kDaprotein,mitochondrial-like	XP_003570534.1	559	100	Cyto	1.7	1.3	1.6	1.3	0.8	1.6
						0.3	1.5	0.8	0.7	1.1	0.5
165	Heat shock cognate 70 kDa protein 2-like	XP_003578898.1	233	100	Cyto	1.3	0.3	0.3	0.3	1.9	1.2
						0.4	1.7	0.4	0.3	0.3	0.7
166	Heat shock cognate 70 kDa protein 2-like	XP_003578898.1	441	99.678	Cyto	1.2	0.7	0.9	1.4	1.5	1.1
						0.1	0.7	1.4	1.9	1.2	0.9
264	Protein disulfide-isomerase-like isoform 2	XP_003577737.1	598	99.998	ER	1.1	0.9	0.4	0.6	1.0	2.1
						0.6	0.5	0.7	0.7	1.6	2.1
272	Heat shock 70 kDa protein, mitochondrial-like	XP_003570534.1	368	100	Cyto	0.7	0.6	0.8	0.5	1.5	0.6
						0.9	0.9	0.4	0.4	0.6	0.4
354	Denticleless protein homolog A-like	XP_003559543.1	67	100	Nucl	0.7	0.3	0.7	1.5	0.5	0.8
						0.5	0.9	0.5	0.0	0.4	0.0
492	Protease 2-like	XP_003560292.1	408	100	Cyto	0.5	0.3	0.5	0.6	1.0	0.7
						0.0	0.3	0.4	1.2	0.5	1.2
**Detoxification and stress fense**											
7	Superoxide dismutase [Cu-Zn]	XP_003574947.1	389	99.998	Cyto	0.6	0.7	0.4	0.9	0.7	0.9
						0.7	0.6	1.2	0.8	0.9	1.1
8	Superoxide dismutase [Cu-Zn] 4A-like	XP_003574947.1	71	100	Cyto	0.8	0.8	0.3	0.8	0.9	1.1
						0.7	0.9	0.9	0.9	1.0	1.0
21	2-Cys peroxiredoxin BAS1	XP_003575085.1	327	100	Cyto	0.8	0.7	0.0	0.9	1.2	0.9
						1.0	0.7	0.8	1.1	1.0	0.8
22	2-Cys peroxiredoxin BAS1	XP_003575085.1	350	100	Plastid	1.0	1.0	1.7	1.4	0.9	1.0
						1.1	0.9	1.8	1.1	0.9	0.7
25	probable glutathione S-transferase GSTU6-like isoform 3	XP_003580395.1	125	100	Cyto	0.4	0.6	1.6	1.0	0.7	1.0
						0.8	0.4	1.0	0.9	0.9	0.9
27	glutathione S-transferase DHAR2-like isoform 1	XP_003569014.1	147	100	Cyto	0.8	1.0	0.9	0.8	0.8	1.3
						1.1	1.0	0.8	1.1	1.1	1.2
30	glutathione S-transferase DHAR2-like isoform 1	XP_003569014.1	245	100	Cyto	0.6	1.2	0.0	2.8	0.0	3.9
						1.6	1.0	2.2	2.0	2.3	2.7
34	Glutathione S-transferase 1-like	XP_003569881.1	532	100	Cyto	2.3	2.6	1.6	3.5	1.7	1.6
						1.4	1.9	2.6	3.1	1.5	2.0
37	L-ascorbate peroxidase 2, cytosolic-like	XP_003562395.1	90	100	Cyto	0.9	1.1	0.7	1.0	1.0	1.4
						0.7	0.9	1.3	0.9	0.8	0.7
38	L-ascorbate peroxidase 1, cytosolic-like	XP_003558178.1	574	100	Cyto	1.3	1.3	1.9	1.1	1.1	1.2
						1.1	1.0	1.1	2.5	1.8	1.1
39	L-ascorbate peroxidase 1, cytosolic-like	XP_003558178.1	109	100	Cyto	0.7	1.2	0.8	0.6	0.7	0.9
						0.9	0.7	0.6	1.0	0.7	1.0
273	probable nucleoredoxin 1-1-like	XP_003562828.1	150	100	Cyto	1.0	0.6	0.4	0.6	0.9	1.3
						0.4	0.8	1.4	1.6	1.4	0.9
398	delta-1-pyrroline-5-carboxylate dehydrogenase 12A1, mitochondrial-like	XP_003568012.1	285	100	Mito	0.0	1.7	0.8	2.0	0.8	1.6
						1.6	1.0	0.0	1.2	2.5	0.3
588	L-ascorbate peroxidase 1	XP_003558178.1	583	100	Cyto	1.4	2.1	1.2	2.4	0.8	0.8
						1.5	1.3	1.0	1.6	0.9	0.6

^a^Spot number as given in Fig. 4.

^b^Accession number: according to the NCBI database.

^c^Protein Score: statistical probability of true positive identification of the predicted protein calculated by MASCOT with 0.3 peptide tolerance and one allowed missed cleavage.

^d^Protein Score C.I.%: the PMF score percentage of protein sequence (Confidence interval: Protein Score C.I.% ≥ 95%).

^e^Cyto, cytoplasm; plastid; Mito: mitochondria; Nucl: nuclear; ER: endoplasmic reticulum.

^f^Average %vol ratio: the average %vol of control is set to 1.

**Figure 6:**
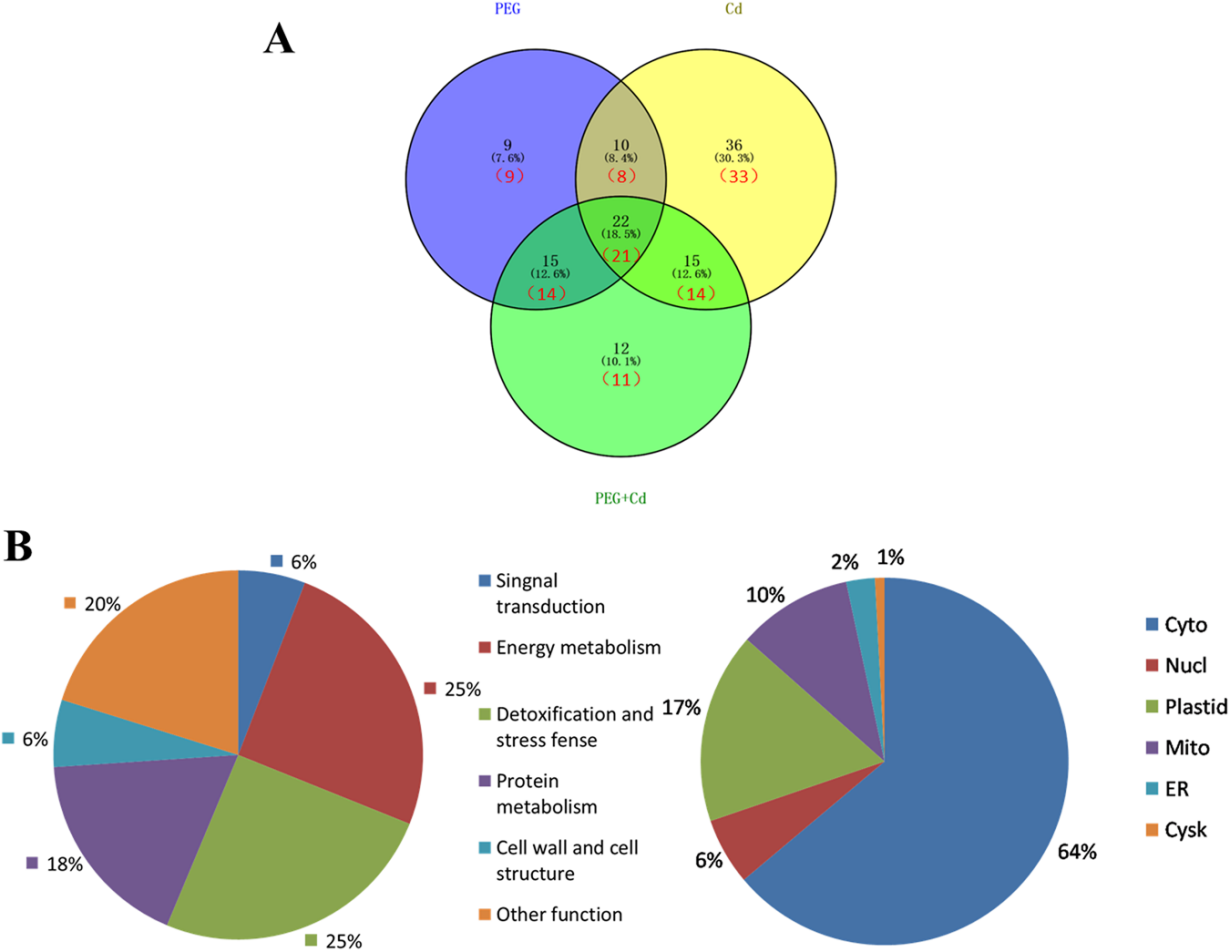
Venn diagram, functional classification, and subcellular localization of 119 identified DAP spots from Bd21 seedling roots under osmotic, Cd^2+^, and their combined stresses after 1, 2, 3 and 4 days. (**A**) Venn diagram analyses of DAP spots. % and number in bracket represent the percentage of DAP spots and unique proteins, respectively. (**B**) (a) Functional classification. (b) Subcellular localization. Labels: Nucl, Nuclear; Cyto, Cytoplasm; Cysk, Cytoskeleton; Mito, Mitochondria; ER, endoplasmic reticulum.

The identified DAP spots and their overlap following the three stress treatments are shown with a Venn diagram (Fig. 6A). Cd^2+^ stress induced the most DAPs (36 protein spots representing 33 unique proteins and accounting for 30.3%) among the three stress treatments. There were 21 proteins commonly present under the three stresses and 9, 33, and 11 proteins specifically induced by PEG and Cd^2+^ stress and their combined stresses, respectively. A comparison between two stress treatments showed that 29 common proteins were induced by the PEG and Cd^2+^ stresses, whereas 23 and 47 were specifically induced by PEG and Cd^2+^ stress, respectively. PEG and the combined stress induced 35 common proteins and 17 and 25 specific proteins, respectively. In addition, Cd^2+^ and the combined stress induced 35 common proteins and 41 and 25 specific proteins, respectively. In particular, the most DAPs occurred at 4 days of Cd^2+^ stress among the three treatments. These results were consistent with those from PCA (Fig. 5) as well as phenotypic, physiological, and ultrastructural analyses (Figs 1–3).

All 119 identified DAP spots were classified into six functional classes (Fig. 6B): energy metabolism (25%), detoxification and stress defense (25%), protein metabolism (18%), signal transduction (6%), cell well and cell structure (6%), and other functions (20%). The subcellular localizations predicted using WoLF PSORT, Predotar, and UniprotKB showed that 64% of the DAPs were located in the cytoplasm, 17% in the plastid, and 10% in the mitochondria (Fig. 6C).

(a) Spot number as given in Fig. 4. (b) Accession number: according to the NCBI database. (c) Protein Score: statistical probability of true positive identification of the predicted protein calculated by MASCOT with 0.3 peptide tolerance and one allowed missed cleavage. (d) Protein Score C.I.%: the PMF score percentage of protein sequence (Confidence interval: Protein Score C.I.% ≥ 95%). (e) Cyto, cytoplasm; plastid; Mito: mitochondria; Nucl: nuclear; ER: endoplasmic reticulum. (f) Average %vol ratio: the average %vol of control is set to 1.

### Accumulation patterns of DAPs under independent and combined stress treatments

**Figure 7:**
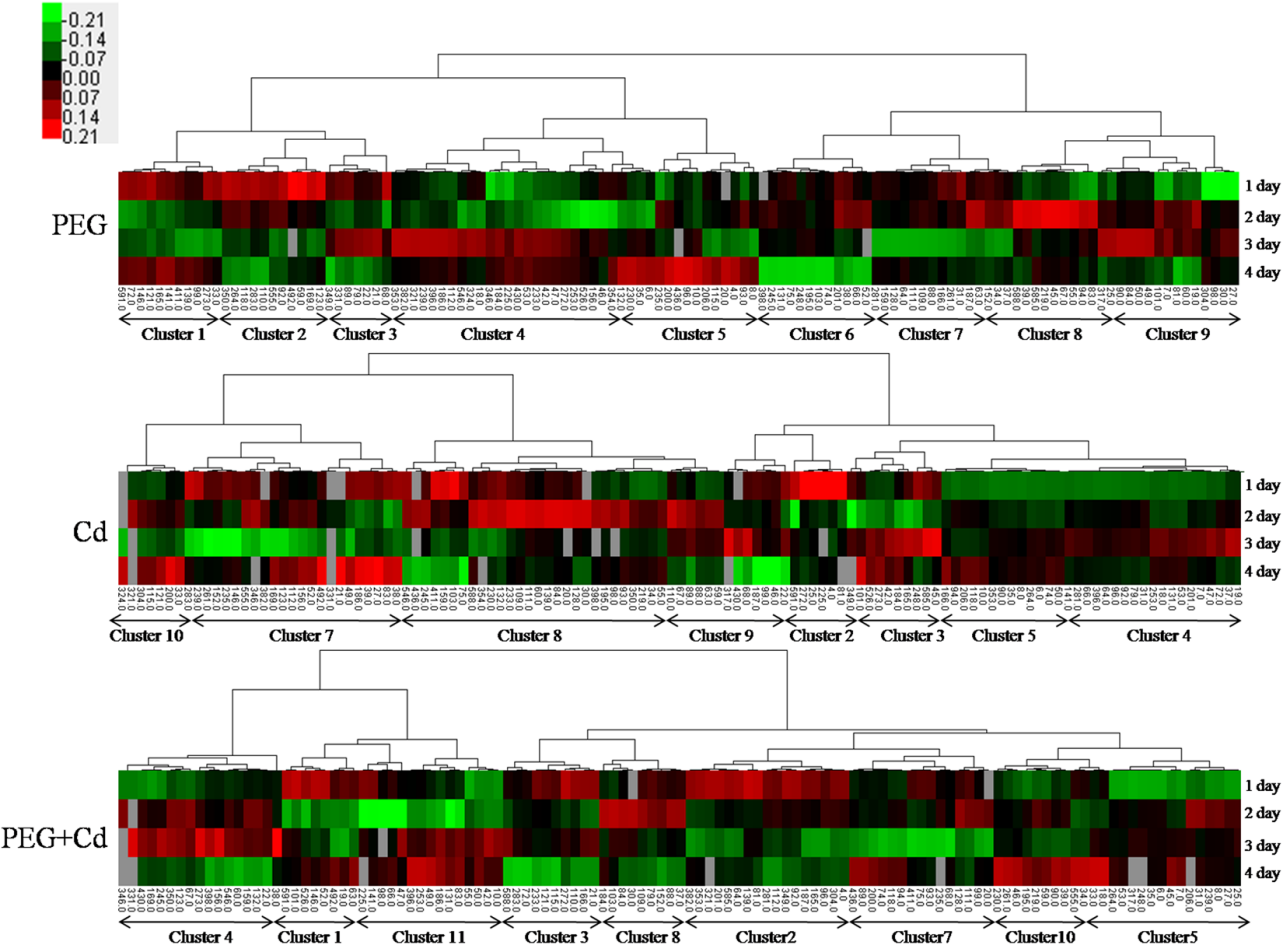
Hierarchical cluster analysis of all identified DAP spots in Bd21 seedling roots.

To visualize the coordinately regulated DAP spots, hierarchical clustering was used to analyze the proteome dataset and hot-maps were constructed to present their dynamic accumulation patterns from 1 to 4 days under the three different stress treatments. As shown in Fig. 7, in response to the three stresses 119 DAP spots could be divided into 11 clusters. Cluster 1 showed a downregulation at day 2, followed by an upregulation at day 3, whereas cluster 2 was downregulated after day 1. Cluster 3 displayed a down-up-down accumulated pattern, whereas cluster 4 showed the opposite up-down pattern. Cluster 5 exhibited an upregulation with a peak accumulation at day 4. Similar to cluster 2, cluster 6 showed a downregulation and declined to a nadir at day 4. Similarly, cluster 7 showed a downregulation and reached the bottom at day 3, then showed an upregulated accumulation at day 4. Similar to cluster 4, clusters 8 and 9 had an up-down accumulation, with a peak difference at 2–3 days after stress treatment. The DAPs in cluster 10 and 11 displayed a down-up accumulation similar to cluster 1, but declined to their lowest point on day 3 and day 2, respectively.

Under PEG osmotic stress, the DAPs showed nine accumulation patterns from cluster 1–9, and lacked cluster 10–11. In total, 107 DAP spots showed an upregulated accumulation, of which 61 occurred at early stages (day 1–2) and 46 at later stages (day 3–4) of stress treatment. Generally, the upregulated DAPs were mainly involved in detoxification and stress defense, energy metabolism, protein metabolism, and signal transduction. The DAPs under Cd^2+^ stress had eight accumulation patterns without cluster 1, 6, and 11. In total, 117 DAP spots showed an upregulated accumulation, with 80 spots upregulated at an early stage and 37 spots at a later stage. Similar to osmotic stress, the upregulated DAPs in response to Cd^2+^ stress were mainly involved in energy metabolism, detoxification and stress defense, protein metabolism, and signal transduction. Compared with PEG osmotic stress, more DAPs showed an upregulated accumulation at early treatment stages, indicating that the root proteome was sensitive to Cd^2+^ stress. The combined stress treatments induced nine expression patterns and lacked cluster 6 and 9. In total, 115 DAP spots showed an upregulated accumulation, and 89 of these DAP spots occurred at days 1–2 (including 55 at day 1) and 26 occurred at days 3–4 of stress treatment. Compared with independent PEG and Cd^2+^ stress, 56 DAP spots had the highest accumulation on day 1 of treatment with combined stress, much more than those from individual stresses.

### Protein–protein interaction (PPI) analysis

Proteins do not perform their functions in isolation within cells; instead they act together in the context of networks^35^. The interaction of different DAPs, which represented by eukaryotic orthologous groups (KOGs), was shown through the PPI network using STRING to reveal potential information at the protein level. The KOGs was obtained through the eggNOG (http://eggnog.embl.de/version_3.0/) database by searching the amino acid sequences of proteins. The amino acid sequences of proteins were downloaded on NCBI (National Center for Biotechnology Information) by searching their GI numbers. To improve the reliability of PPI analysis, the confidence score was set to 0.80. Then the PPI network was displayed using the Cytoscape (version 3.0.0, http://www.cytoscape.org) software. The PPI network was constructed to highlight interactions and relationships between different proteins (Fig. S3). A considerable number of DAPs identified in roots under the three stress treatments were involved in energy metabolism, protein metabolism, signal transduction, cell well and cell structure, detoxification, and stress defense. KOG0841, which represents two 14-3-3 proteins (GF14-B and GF14-D), and KOG0279, which represents guanine nucleotide-binding protein subunit beta-like protein A (RACK1A), were both classified as signal transduction-related proteins. In particular, 14-3-3 proteins interacted with many proteins involved in signal transduction, energy metabolism, detoxification and stress defense, protein metabolism, and cell structure, indicating their important roles in response to abiotic stresses.

### Dynamic transcriptional expression analysis of key DAP genes by quantitative real-time polymerase chain reaction (qRT-PCR)

**Figure 8:**
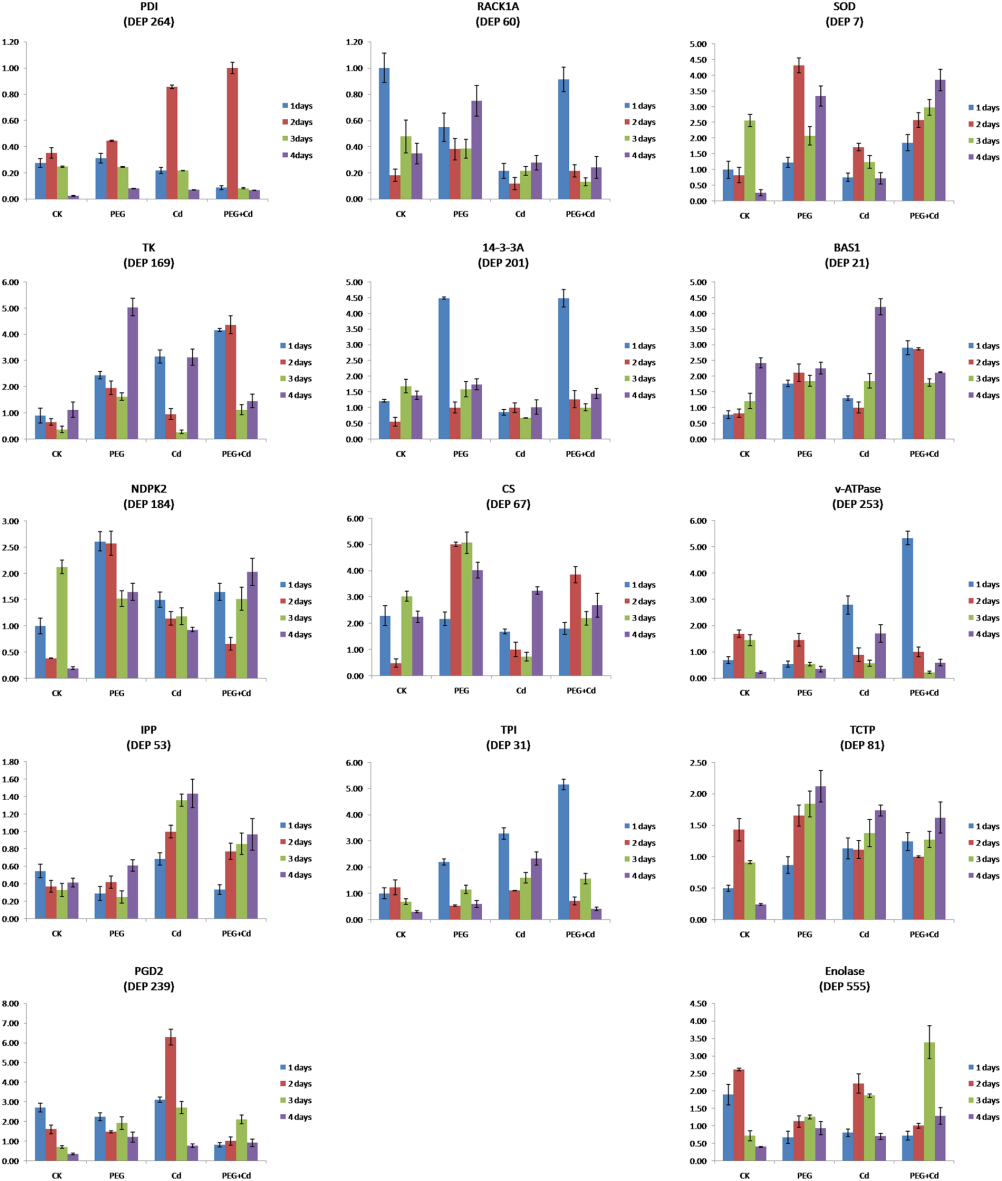
Dynamic transcriptional expression changes of fourteen key DAP genes in Bd21 seedling roots under PEG, Cd^2+^and their combined stresses as revealed by qRT-PCR.

According to proteome and PPI analyses, 14 key DAPs (nodes with green edges in Fig. S3) were selected for further investigation of their transcript expression patterns under the three stress treatments (Fig. 8). Among them, three DAP genes (RACK1A, NDPK2, and 14-3-3 A) were involved in single transduction, two (CS and PDI) were related to protein metabolism, two (SOD and BAS1) participated in detoxification and stress defense, and seven (TPI, IPP, TCTP, TK, PGD2, v-ATPase, and Enolase) were related to energy metabolism. The specific primers are listed in Table S4, and their specificities were verified by observing the melting curve of the RT-PCR products and the specific band on the agarose gel (Fig. S4).

The results showed that two DAP genes (TK and PGD2) displayed a high consistency under osmotic stress, two genes (BAS1 and 14-3-3A) displayed a high consistency under Cd^2+^ stress, and two genes (SOD and v-ATPase) displayed a high consistency under the combined stress. A similar trend between transcriptional and translational expression patterns was observed for six genes (SOD, IPP, CS, TCTP, 14-3-3A, and PDI) under osmotic stress, six genes (SOD, IPP, TCTP, NDPK2, PDI, and Enolase) under Cd^2+^ stress, and seven genes (BAS1, CS, TCTP, TK, 14-3-3A, PGD2, and PDI) under the combined stress. The remaining genes showed a poor consistency between their transcription and translation levels under different treatments (Fig. 8 and Table S2). These results indicated that both transcriptional and translational regulations were involved in the abiotic stress response in seedling roots.

## Discussion

In this study, we identified a considerable number of DAPs in response to PEG, Cd^2+^, and their combined stresses in Bd21 seedling roots, which participated in wide metabolic processes. Here, we further discuss their metabolic pathways and potential functions involved in the abiotic stress response and defense of plants.

### Signal transduction

Abiotic stresses can change the expression of several signal transduction-related proteins. In higher plants, the activities of many signal transduction-related proteins are regulated by the 14-3-3 family of proteins. Thus, the 14-3-3 family proteins can play important roles in plant abiotic and biotic stress responses and defense^36^. Studies from rice have shown that the 14-3-3 family is responsive to various biotic and abiotic stresses^37,38^. The 14-3-3 proteins may regulate various biochemical processes by interacting with more than 200 target proteins^39^. They act within protein phosphorylation cascades by associating with specific phospho-sites^40^, altering enzyme activity, preventing or inducing protein degradation, or affecting the subcellular localization of proteins by binding to their targets^41,42^. In addition, 14-3-3 proteins can activate Ca^2+^-dependent protein kinase (CDPK)^43^, which in turn can activate a variety of transcription factors that regulate gene expression^44^. Moreover, 14-3-3 proteins can enhance tolerance of osmotic stress through higher transpiration and photosynthesis rates caused by increased opening of the stomata^45^ and allocating more carbon from the shoot to the root as well as enhancing proton secretion in the root growing zone^46^. 14-3-3 proteins can interact with and activate the ROS-mediated mitogen-activated protein kinase (MAPK), which can scavenge extra ROS caused by Cd stress^47^.

Our recent study has found that 14-3-3A was upregulated under salt stress at both the transcriptional and translational levels in seedling leaves of *B*. *distachyon*, and the 14-3-3-like protein GF14-B was phosphorylated under salt stress treatment^48^. In addition, the 14-3-3-like proteins GF14-B and GF14-D were downregulated in *Brachypodium* seedling roots under H_2_O_2_ stress^49^. Furthermore, transcriptional expression analysis of the BdGF14 gene indicated that the 14-3-3 genes are significantly regulated by various abiotic stresses^47^. In this study, we identified six DAPs related to signal transduction in the roots under three stress treatments, including three 14-3-3 family proteins (14-3-3-like protein A, GF14-B, and GF14-D). These proteins showed significant changes in accumulation in response to the three stress treatments (Table 1). The PPI network revealed that 14-3-3 proteins interacted with many proteins involved in signal transduction, energy metabolism, redox homeostasis and stress defense, and protein folding and degradation (Fig. S3), suggesting their central roles in response to abiotic stresses. In particular, 14-3-3-like protein A (spot 201) and GF14-B (spot 206) displayed an upregulated accumulation, whereas GF14-D (spot 74) displayed a downregulated accumulation under osmotic, Cd^2+^, and their combined stresses (Table 1 and Fig. 7), indicating their crucial roles in the plant abiotic stress response and defense through signal transduction.

Guanine nucleotide-binding protein subunit beta-like protein A (spot 60), also known as receptor for activated C kinase 1A (RACK1A), is a WD-40-type scaffold protein and is conserved in eukaryotes. RACK1A, which is the major component of the RACK1 regulatory proteins, plays a role in diverse signal transduction and stress response pathways by interacting with multiple proteins to modulate molecular responses^50^. This protein showed an upregulated accumulation after 2–3 days of the three stress treatments (Table 1). We also found that NDPK2 (spot 184) was downregulated under the three stress treatments. In addition to its basic function in phospho-transfer and regeneration of nucleoside triphosphates, NDPK2 is involved in several signaling pathways including phytochrome, auxin signaling, heat shock responses, and H_2_O_2_ signaling, which may be related to the H_2_O_2_-mediated mitogen-activated protein kinase signaling cascade^51^.

### Redox homeostasis and stress defense

Higher plants have evolved a series of systems to scavenge ROS during seedling growth, which can help plants survive when suffering from various adverse environments such as osmotic and heavy metal stresses. Nucleoredoxin 1-1 serves as a redox sensor to regulate the Wnt/β-catenin signaling pathway, which control cell proliferation, cell fate, and utilization of ROS^52,53^. It was down-up-accumulated under osmotic stress, up-accumulated under Cd^2+^ stress, and up-down-accumulated under combined stress (Fig. 7). Glutathione S-transferase (GST, spots 25, 27, 30, and 34) plays crucial roles in both normal cellular metabolism and in detoxification of a wide variety of xenobiotic compounds^54^. It also plays a significant role in the ascorbate recycling system and is important in maintaining redox homeostasis as well as in the scavenging of ROS during oxidative stress defense^55^. We found that four GST protein spots generally showed a significantly upregulated accumulation under the three stress treatments (Table 1 and Fig. 7). In particular, the abundance of spot 30 was increased almost four-fold after 2 days of combined stress treatment (Table 1). The significant upregulation of these protein spots could scavenge extra ROS and improve plant cellular metabolism, detoxification, and stress defense.

APX1 (spots 38, 39, and 588) and APX2 (spot 37) are key enzymes involved in the removal of ROS^56^. The accumulation patterns showed that APX protein spots 17 and 18 were generally upregulated under osmotic, Cd^2+^, and combined stresses and spot 588 displayed a significant upregulation under both PEG osmotic and Cd^2+^ stresses (Table 1). 2-Cys peroxiredoxin BAS1 (BAS1, spots 21 and 22) plays a key role in regulating the level of H_2_O_2_, takes part in signal transduction as an antioxidant, and contributes to the reduction of ROS detoxification^12,57^. It was upregulated under Cd^2+^ or combined stress. Superoxide dismutase (Cu-Zn) (SOD, spots 7 and 8), an important antioxidant enzyme, can modify the toxic and highly reactive superoxide anion and forms the less harmful hydrogen peroxidein in mitochondria. Hydrogen peroxide is later cleared by catalase to achieve detoxification and defense against the stress^58^. Its response to the three stress treatments was similar to BAS1 (Table 1). The upregulated accumulation of these stress defense proteins could protect plant cells from the lethal effects of ROS under osmotic and Cd^2+^ stress, as well as their combined stresses. Moreover, significantly changes of MDA, proline and betaine also reflect the response of roots to abiotic stresses.

### Protein folding and degradation

Proteins were misfolded and lost normal assembly and function under abiotic stresses. These non-functioning proteins were refolded and degraded. Molecular chaperones are proteins that participate in the covalent folding or unfolding and the assembly or disassembly of other macromolecular structures. Molecular chaperones are present when the macromolecules perform their normal biological functions and have been correctly folded and/or assembled. Heat shock 70 kDa protein (HSP70, spots 159, 165, 166, and 272) acts as a molecular chaperone in all organisms and is important for various essential cellular processes such as protein folding and protein transport across membranes^59,60^. We found that the accumulation of HSP70 was generally upregulated under independent and combined stresses (Table 1 and Fig. 7). In our recent study, HSP70 was significantly upregulated in roots at 4 and 6 h under H_2_O_2_ stress^49^. This could improve protein folding and transport in plants subjected to adverse environments. Another molecular chaperone, protein disulfide-isomerase-like isoform 2 (PDI, spot 264) has an athioredoxin (TRX) domain structure and contributes to the formation of proper disulfide bonds during protein folding^61^. In this study, this protein had a clear upregulated accumulation under combined PEG osmotic and Cd^2+^ stresses, and its transcript expression level was also dramatically upregulated under Cd^2+^ and combined stresses (Fig. 8). Our recent study showed that PDI is maintained at a high level in roots between 24 and 48 h of drought stress treatment^62^. Thus, PDI could function in normal disulfide bond formation and protein folding in response to abiotic stresses.

Denticleless protein homolog A (DTL, spot 354) is a component of the DCX (DTL) E3 ubiquitin ligase complex, which is related to cell cycle control, the DNA damage response, and DNA synthesis^63,64^. We found that this protein was generally downregulated under the three stresses, but with a clear upregulation after 2 days of treatment, indicating it plays a role in the heavy metal stress response. The 40 S ribosomal protein S7 (spot 18), which forms the ribosome, is crucial in protein synthesis and was upregulated in response to drought treatment in roots^62^. In this study, it was upregulated at 1–2 days under osmotic and Cd^2+^ stresses, and significantly upregulated under the combined stress. This indicated that protein synthesis in seedling roots was stimulated by abiotic stresses. The increased protein metabolism in seedling roots under osmotic, Cd^2+^, and combined stresses is essential for plant adaptation to abiotic stresses^65^.

### Energy metabolism

Energy metabolism, including glycolysis and the tricarboxylic acid cycle, mainly provides the energy for plant growth and development^66^. The ATPase beta subunit (spot 526) and ATP synthase 24 kDa subunit (spots 45 and 331) are ATP enzyme subunits. These subunits showed clear upregulation at different time points of the three stress treatments (Table 1), hinting that ATP synthesis was active under abiotic stresses. Malate dehydrogenase (MDH, spot 92) can remarkably enhance salt and aluminum toxicity resistance of plants by increasing the levels of malic acid^67,68^. A recent study showed that MDH1 was significantly accumulated under Cd^2+^ stress^69^. In this study, MDH was downregulated at 1–3 days, but upregulated at day 4 under the three stresses, indexing that MDH could play a role in plant response and adaptation to abiotic stresses. Glyceraldehyde-3-phosphate dehydrogenase (GAPDH, spot 72) and triosephosphate isomerase (TPI, spots 31 and 33), combined with MDH, were conducive to triosephosphate synthesis, which promotes energy production^70^. Both GAPDH and TPI were significantly upregulated under the three stress treatments (Table 1 and Fig. 7). TPI in roots showed a rapid response to abiotic stresses and its transcript level was dramatically upregulated at day 1 following the three stress treatments (Fig. 8). This is consistent with our recent study showing that GAPDH and TPI were upregulated in leaves under osmotic and Cd^2+^ stress and the combined stress^69^. Thus, energy provision is important for plant adaption and defense against abiotic stresses.

Enolase (NSE, spot 555), a key glycolytic enzyme responsible for the catalysis of the conversion of 2-phosphoglycerate (2-PG) to phosphoenolpyruvate (PEP), the ninth and penultimate step of glycolysis, plays an important role in the energy metabolism of cells^71^. A recent study indicated that NSE was upregulated under drought stress in wheat leaves^12^. In our study, NSE showed significant accumulation under the three stresses: 3.4-fold upregulation on day 1 of PEG treatment, 5.4-fold upregulation on day 2 of Cd^2+^ treatment, and 5.9-fold upregulation on day 4 of combined treatments (Table 1). Its transcript expression also showed a peak at 1 day of combined stress treatment (Fig. 8). Phosphoglucomutase (PGM, spot 152) in the cytoplasm (cPGM), widely distributed in plant cells, participates in metabolic processes such as photosynthesis, respiration, and cell wall synthesis. A previous study indicated that the growth of *Arabidopsis* was significantly inhibited after loss of cPGM activity^72^. Our recent study displayed that PGM was upregulated under both osmotic stress, Cd^2+^ stress, and combined stress in *Brachapodium* leaves^69^. In this study, when roots were subjected to the three stresses, PGM was upregulated. Therefore, PGM upregulation induced by PEG osmotic stress, Cd^2+^ stress, and the combined stress could enhance plant adaptation and defense against abiotic stresses.

### A putative metabolic pathway of the *Brachapodium* seedling root response to osmotic, Cd^2+^, and combined stresses

**Figure 9:**
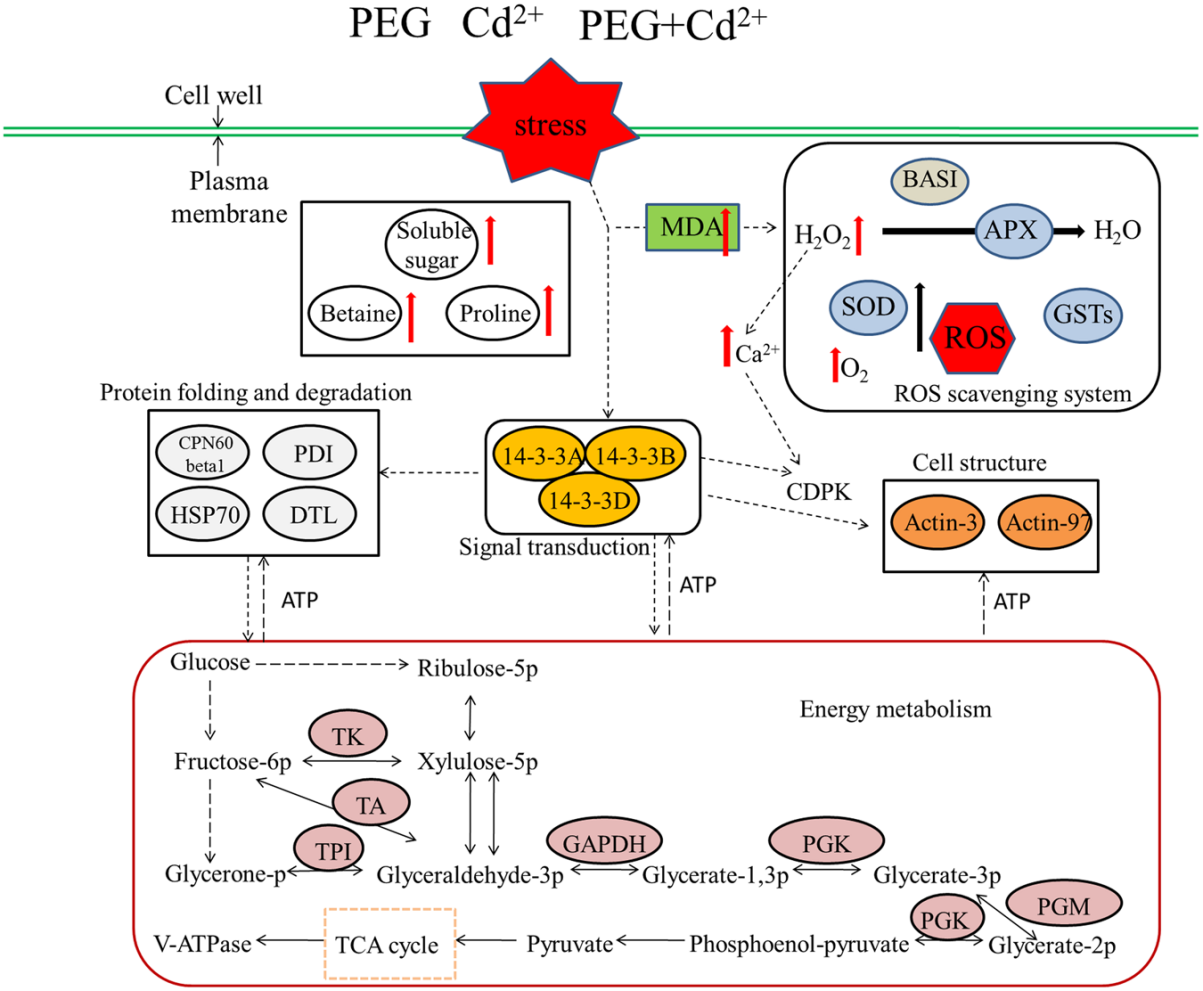
An overview of metabolic pathways of Bd21 seedling roots under osmotic, Cd^2+^, and their combined stresses.

Here, we proposed a putative metabolic pathway of the *Brachapodium* seedling root response to PEG osmotic stress, Cd^2+^ stress, and their combined stress based on the comparative proteome analysis and previous findings, which provided an overview of proteome changes under independent and combined abiotic stresses (Fig. 9). When seedling roots suffered from single and combined osmotic and Cd^2+^ stresses, cells of the roots produced stress signals to cause differential accumulation of signal transduction-related proteins. Particularly, 14-3-3 proteins activated CDPK^44^, which activates transcription factors that induce or repress gene expression. Along with the extension of stresses, MDA and ROS quickly accumulated in cells, which induced a series of phenotypic, physiological, and proteomic changes and the soluble sugar, betaine, and proline contents were significantly increased. Simultaneously, the increased accumulation of H_2_O_2_ resulted in rapid accumulation of Ca^2+^ in the cytosol^73^, which promoted the activation of CDPK^74^. With the increase in ROS, the abundance levels of BAS1, APX, SOD, and GSTs were increased and the ROS scavenging system was activated to reduce ROS and maintain normal intracellular oxidation-reduction levels. Meanwhile, increased accumulation of molecular chaperone proteins such as CNP60 and HSP70 reduced misfolding or improper assembly of proteins and maintained protein function. Significantly increased metabolism could maintain the energy supply in root cells to generate ATP and enhance the ability of plants to adapt and resist abiotic stresses.

## Conclusion

PEG osmotic stress, Cd^2+^ stress, and their combined stresses caused inhibition of seedling growth and significant phenotypic and physiological changes, including decreases in fresh water, plant height, main root length, and relative water content, and increases in proline, MDA, soluble sugar, and betaine contents, as well as abnormal cell ultrastructures. Particularly, Cd^2+^ and combined osmotic and Cd^2+^ stresses had more severe effects on seedling growth than independent stress. In total, 106 unique DAPs were identified by 2D-DIGE combined with 2-DE and tandem mass spectrometry, which were mainly involved in energy metabolism, detoxification and stress defense, and protein metabolism. A 14-3-3-centered sub-network revealed by PPI analysis indicated that 14-3-3 proteins play important roles in response to different abiotic stresses. Moreover, qRT-PCR analysis revealed that both transcriptional and translational regulations were involved in abiotic stress responses in seedling roots. These findings provide new insights into the molecular mechanisms of plant responses to and defense against abiotic stresses.

## Materials and Methods

### Plant materials and stress treatments

Seeds of *Brachypodium distachyon* L. (Bd21), kindly provided by Dr. John Vogel from USDA-ARS, were surfacely sterilized in 5% sodium hypochlorite for 5 min, and rinsed 4 times in sterile distilled water. Seeds were submerged in water for 12 h at room temperature, and then transferred to wet filter paper for 24 h to germinate at room temperature (22–25 °C). The uniformly germinated seeds were selected to grow in plastic pots containing Hoagland solution that was changed every two days. At the three leaf stage, the seedlings were treated by osmotic stress (15% polyethylene glycol PEG6000), Cd^2+^ (500 μMCdC1_2_) and their combination (15% PEG6000 + 500 μM CdC1_2_) in three biological replicates. The samples before treated with PEG and CdC1_2_ were used as a control. After treatments for 1 day, 2 days, 3 days, 4 days, 5 days, 6 days, and 7 days, the roots of seedling and the seedlings from all of the biological replicates were used to measure physiological indicators. There maining root samples were snap-frozen in liquid nitrogen and then stored at−80 °C prior to use for later RNA and protein extraction and proteome analysis.

### Physiological parameters measurement and TEM observation

The roots were used to measure the physiological parameters, including the length of the main root, plant height, fresh weight and dry weight of whole seedling. Relative water content (RWC) was measured according to Lv *et al*.^48^. Malondialdehyde (MDA) and soluble sugar contents were measured by using reagent kit from the Nanjing Jiancheng Bioengineering Institute of Jiangsu Province, China (Cat. no. A084-3 and A003-1). Root free proline content was measured according to Bates *et al*.^75^ with minor modifications. Approximately 0.5 g of samples was homogenized with 5 ml of 3% (w/v) aqueous sulfosalicylic acid solution. The homogenate was centrifuged at 3000 g for 20 min. The supernatant, acid–ninhydrin agent and glacial acetic acid (2 mL each) were mixed and boiled for 1 h. The reaction mixture was extracted with 4 mL toluene. Then the homogenate was centrifuged at 3000 g for 10 min. The absorbance at 520 nm was determined via using L-proline as a standard. Proline content was expressed in micrograms per gram per unit fresh weight. Betaine content was measured by using reagent kit from the Suzhou Comin Biotechnology Co., Ltd of Jiangsu Province, China (Art. No. TCJ-1-G), and the experiments were conducted in strict accordance with the manufacturer’s instructions. Three biological replicates were used to minimize experimental error.

For TEM observation of root ultrastructures, the small pieces of sample from tips of roots (2 mm) of 2 days under three abiotic stresses were fixed in 4% (v/v) glutaraldehyde, vacuum-infiltrated until the material sank and left overnight at 4 °C. Then the samples were rinsed with 0.1 M phosphate buffer (pH 7.2) and post-fixed with 1% osmiumtetroxide for 8 h at 4 °C. After fixation, samples were dehydrated in a series of graded alcohol and propylene oxide and embedded in resin and polymerized in thermostatic chamber at 37 °C (12 h), 45 °C (12 h), 60 °C (48 h). Ultrathin sections (70–90 nm in thickness) were generated using a Lycra UC6 ultramicrotome and stained with 2% uranyl acetate for 30 min followed by lead citrate for 15 min. Then the sections were examined on a Hitachi H7500 TEM at 100 kV^76,77^.

### Protein extraction, 2D-DIGE, image acquisition and data analysis

Root total proteins from treatments of 1 day, 2 days, 3 days and 4 days with three biological replicates in each treatment were extracted according to the method of Lv *et al*.^48^. Each sample was extracted three times, and protein concentrations were determined with a 2D-Quant-Kit (GE Healthcare, USA). The differentially accumulated protein (DAP) spots at 2 days were firstly identified by two-dimensional difference gel electrophoresis (2D-DIGE), and then two-dimensional gelelectrophoresis (2-DE) was used to separate the identified DAPs for tandem mass spectrometry and dynamic accumulation analyses at different treatment times. Protein separation consists of protein labeling and 2D-DIGEbased on the published methods in Dong *et al*.^78^, and the details of 2D-DIGE experiments were showed in Table S1. IEF was performed with the Ettan IPG-phor system (GE Healthcare, USA) in immobiline pH gradient Dry Strips (GE Healthcare, pH 3–10, 18 cm, USA).Proteins of 1 mg extracted from each sample were run separately on conventional 2-DE gels for protein identifications. This step was repeated three times for error control.

After 2-DE, the gels were stained with Coomassie Brilliant blue G-250 (CBB; Sigma, USA). The 2-DE images were scanned by GS-800 Calibrated Densitometer and statistical analysis was performed by the Image Master^TM^ 2-D platinum software version 7.0, which allowed spot detection, landmarks identification, aligning/matching of spots within gels, quantification of matched spots according to the manufacturer’s instructions. Spot volume was used as the analysis parameter for quantifying protein accumulation amount. Relative spot volumes (% V) (V = integration of OD over the spot area; %V = V single spot/V total spot) were used for the quantitative analysis in order to decrease experimental errors. Spot volume ratios that showed a statistically significant difference (≥two-fold difference in vol. %*p* < 0.05) by Student’s t-test at one or more treatments were processed for the further analysis.

### Statistical analysis

The coordinately regulated DAP spots were used to perform hierarchical clustering analysis by Cluster 3.0 software according to the method described by Eisen *et al*.^79^. The relative ratios of DAP spots were conducted log 2 transforming, and then the Euclidean distance similarity metric was used to define the similarity and the hierarchical clusters were assembled using the complete-linkage clustering method. Cluster 3.0 allows for clustering result visualization with a dendrogram of the DAP spots.

Principal component analysis (PCA) is a method that finds the main variations and reveals hidden structures present in the data set^80^. In this study, coefficient and KMO and Bartlett’s test of sphericity were selected for dimension reduction analysis and the results were displayed in the loading plot and the scatter plot, respectively. The loading plot and scatter plot of PCA was calculated or displayed with the average center value of each DAP spot. PCA was performed using SPSS 19.0 software.

### Protein identification using MALDI-TOF/TOF-MS and database searching

The selected protein spots were manually excised from the 2-DE gels and digested with trypsin. Identification of the spots was performed by using matrix-assisted laser desorption/ionization time-of-flight/time-of-flight mass spectrometry (MALDI-TOF/TOF-MS). The MS/MS spectra were searched against the NCBI *Brachypodium* protein database (47737 entries in total, downloaded on May 10, 2017) using software MASCOT version 2.2 (Matrix Science) with the following parameter settings: Trypsin cleavage, one missed cleavage allowed, Carbamidomethyl (C) set as fixed modification, Oxidation (M) allowed as dynamical modifications, peptide mass tolerance set to ±100 ppm and fragment tolerance set to ±0.4 Da. All searches were evaluated based on the significant scores obtained from MASCOT. The Protein Score C. I.% and Total Ion Score C. I.% were both set above 95% and the significance threshold *p* < 0.05 for the MS/MS.

### Bioinformatics

Protein function classification was based on the annotation from AgBase version 2.00. Subcellular locations of DAPs were predicted based on the combination of WoLF PSORT (http://wolfpsort.org/), Predotar (https://urgi.versailles.inra.fr/predotar/ predotar.html), and UniprotKB (http://www.uniprot.org/). Then, the sequences of all the DAPs were used for BLAST analysis with the National Center for Biotechnology Information (NCBI) clusters of Eukaryotic Orthologous Groups (KOG) database to obtain the KOG numbers of those proteins by eggNOG (http://eggnog.embl.de/version_4.0.beta/). A data set containing all the KOG numbers was then used for protein-protein interactions (PPI) analysis by using the Search Tool for Retrieval of Interacting Genes/Proteins (STRING) database (version 9.1, http://string-db.org). Only the interactions that had a confidence score of at least 0.8 and were exclusively based on co-expression and experiment conditions were utilized to construct the network, then they were displayed using the Cytoscape (version 3.0.0, http://www.cytoscape.org) software. Furthermore, a putative metabolic pathway was gained using KEGG pathway (www.genome.jp/kegg/pathway.html) databases.

### RNA extraction and quantitative real-time polymerase chain reaction (qRT-PCR)

qRT-PCR was used to detect the dynamic transcript expression changes of the representative DAP genes at different treatment times. Total RNA from the roots of Bd21 at each time points from one to four day treatments was isolated using TRIzol Reagent (Invitrogen). Then genomic DNA was removed and reverse transcription reactions were performed with the PrimeScript® RT Reagent Kit with gDNA Eraser (TaKaRa, Shiga, Japan) according to the manufacturer’s instructions. Gene-specific primers were designed using online Primer3 Plus (http://www.bioinformatics.nl/cgi-bin/primer3plus/primer3plus.cgi) and their specificities were checked by observing the melting curve of the RT-PCR products and the specific band on the agarose gel. qRT-PCR was performed followed the methods described by Lv *et al*.^48^. All qRT-PCR reactions were performed in triplicate and Ctvalues were averaged. The fold changes in the target gene relative to the *B. distachyon* constitutively expressed Ubqutin (UBI) gene were determined by: Fold Changes = 2−Δ (ΔCt), where ΔCt = Ct_target_ − Ct_SamDC_ and Δ(ΔCt) = ΔCt_treated_−  ΔCt_control_, according to the periments (MIQE) guidelines.

## Electronic supplementary material

Supplementary information

Table S2

Table S3

Table S4
