# Supplementary material for: Identification of differentially accumulated proteins involved in regulating independent and combined osmosis and cadmium stress response in Brachypodium seedling roots
Source: Sci Rep. 2018 May 17;8:7790. doi: 10.1038/s41598-018-25959-8 (PMC5958118; doi:10.1038/s41598-018-25959-8)
Supplement: Supplementary file 1 — Supplementary information [file 41598_2018_25959_MOESM1_ESM.pdf]

Identification of differentially accumulated proteins involved in regulating independent and combined osmosis and cadmium stress response in *Brachypodium* seedling roots

Ziyan Chen[1**], Dong Zhu[1**], Jisu Wu[1**], Zhiwei Cheng[1], Xing Yan[2*], Xiong Deng[1] & Yueming Yan[1*]

[1]College of Life Science, Capital Normal University, 100048 Beijing, China

[2]State Key Laboratory of Earth Surface Processes and Resource Ecology, College of Global Change and Earth System Science, Beijing Normal University, 100875 Beijing, China

E-mail:

Chen ZY: chen2821301@163.com

Zhu D: ZD19920804@163.com

Wu JS: wujs1226@126.com

Cheng ZW: HendyCZW@aliyun.com

Yan X: yanxing@bnu.edu.cn

Deng X: dengxiongred@163.com

[*]Corresponding author:

Prof. Dr. Yueming Yan, College of Life Science, Capital Normal University, Xisanhuan Beilu No. 105, 100048 Beijing, P. R. of China.

Tel. and Fax.: 0086-10-68902777

E-mail: yanym@cnu.edu.cn

[**]These authors contributed equally to this work

Supplementary information

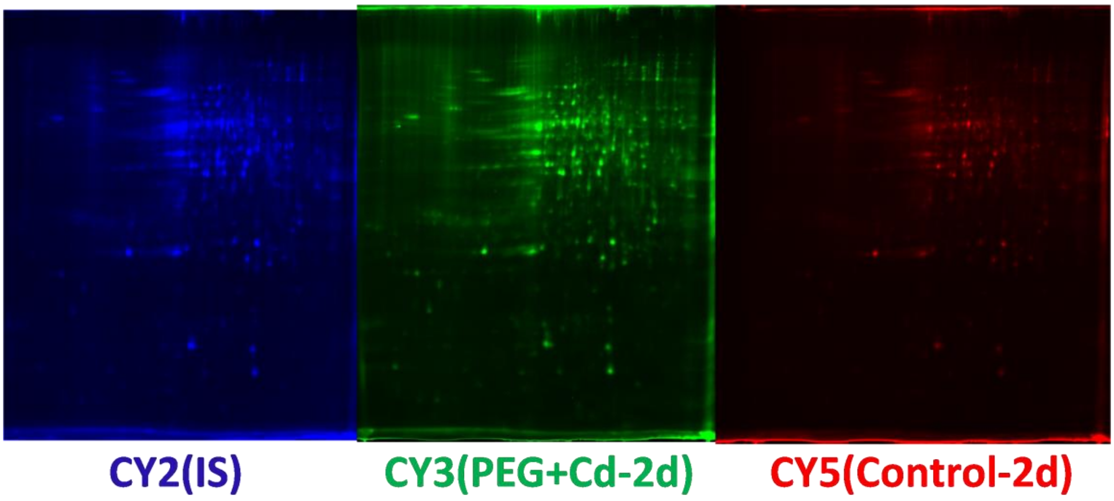

Fig. S1. Cy2, Cy3 and Cy5 images of 2D-DIGE of whole proteins in Bd21 seedling roots.tif

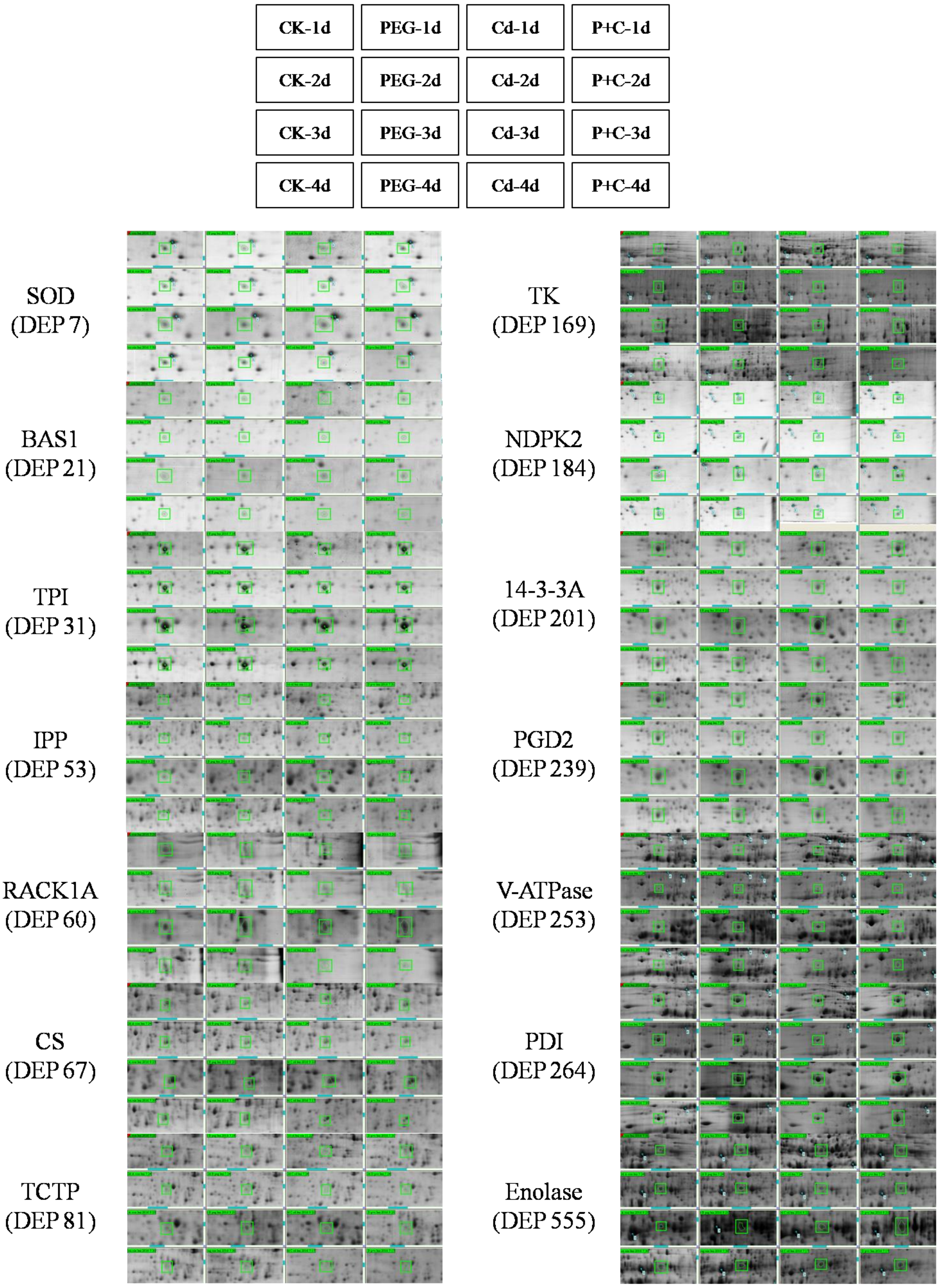

Fig. S2. Fourteen key DAPs of Bd21 seedling roots in the level of proteomics.tif

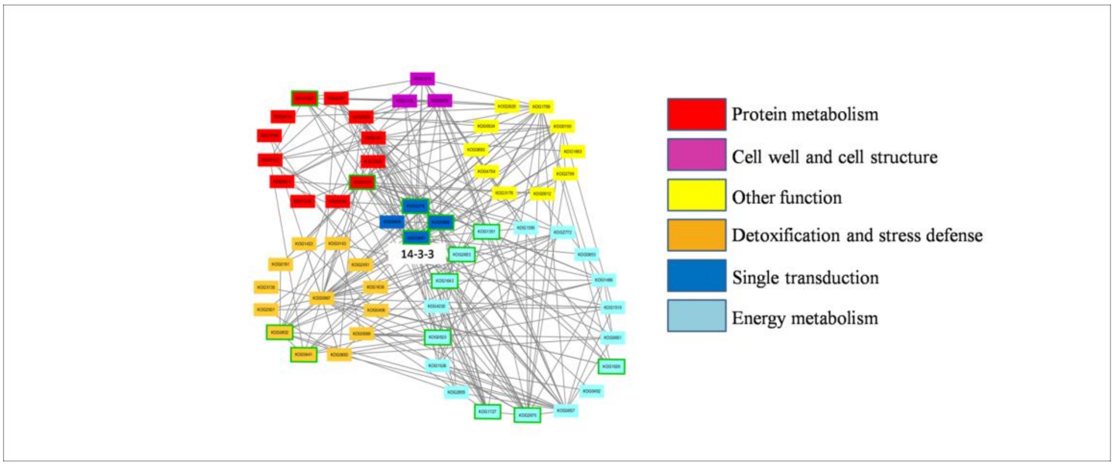

Fig. S3. Network of key DAPs in Bd21 seedling roots involved in osmotic and Cd^{2+} stress adaptation and tolerance. Interactions of the DAPs were extracted by searching the STRING database with a confidence cutoff of 0.80. The interaction network was reconstructed using the Cytoscape software.

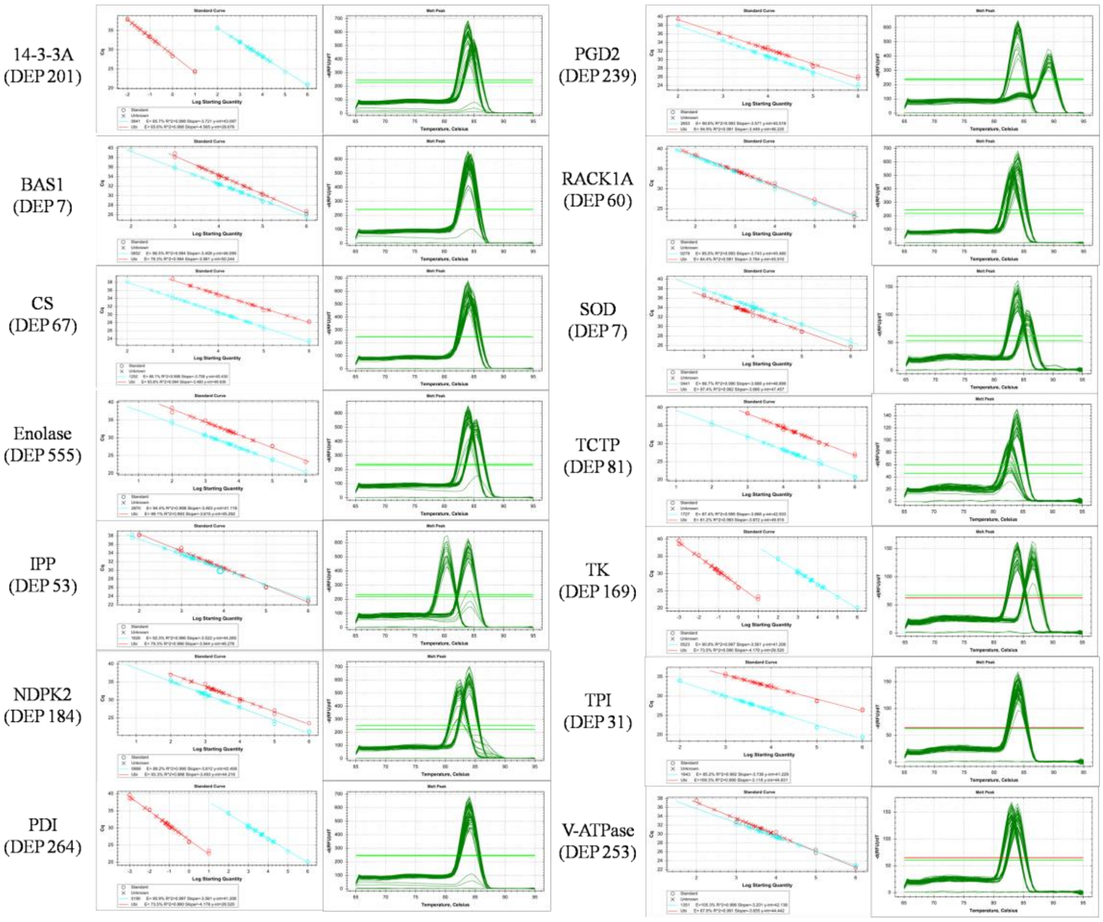

Fig. S4. Double standard curves and dissolution curve of fourteen key DAP genes.tif

Table S1. Details of 2D-DIGE experimental design for detecting differentially accumulated protein*

Gel Number	Cy2 (Internal Standard-Pool)	Cy3 (Sample)	Cy5 (Sample)
Gel 1	CK-2d-1 + PEG-2d-1 + Cd-2d-1 + PEG+Cd-2d-1 + CK-2d-2 + PEG-2d-2 + Cd-2d-2 + PEG+Cd-2d-2 + CK-2d-3 + PEG-2d-3 + Cd-2d-3 + PEG+Cd-2d-3	CK-2d-1	PEG-2d-1
Gel 2		PEG-2d-2	Cd-2d-1
Gel 3		Cd-2d-2	PEG+Cd-2d-1
Gel 4		PEG+Cd-2d-2	CK-2d-2
Gel 5		CK-2d-3	Cd-2d-3
Gel 6		PEG-2d-3	PEG+Cd-2d-3

*Each sample contained three biological replicates.

Table S1. Details of 2D-DIGE experimental design for detecting differentially accumulated proteins.doc

Table S2.Complete list of 119 differentially accumulated protein spots in Bd21 seedling roots under control and three abiotic stress treatments.xls.

a) Spot number as given in Fig. 4. b) Accession number: according to the NCBI database. c) Protein Score: statistical probability of true positive identification of the predicted protein calculated by MASCOT with 0.3 peptide tolerance and one allowed missed cleavage. d) Protein Score C.I.%: the PMF score percentage of protein sequence (Confidence interval: Protein Score C.I.% $\geqslant$ 95%). e) Pep. Count: matched peptide count. f) TpI/TMW (kDa): pI of predicted protein/molecular mass of predicted protein. g) Cyto, cytoplasm; plastid; Mito: mitochondria; Nucl: nuclear; ER: Endoplasmic reticulum. h) Average %vol ratio: the average %vol of control is set to 1.

Table S3. The identified differentially accumulated protein spots from 2-DE maps of Bd21 seedling roots under PEG, Cd^{2+} and their combined stress treatments in combination with MALDI-TOF/TOF-MS data.xls

(a) The statistical probability of a true positive identification of the predicted protein, as calculated by MASCOT using MS and MS/MS data. (b) The matched peptide count.

Table S4. Primer sequences used for qRT-PCR.xls
